# Long-term application of straw and biochar improves soil carbon fractions, enzyme activity, and tomato yield under continuous greenhouse cultivation

**DOI:** 10.3389/fpls.2026.1808305

**Published:** 2026-04-30

**Authors:** Xiaoyang Yu, Zhen Wang, Meiqi Guo, Tianqi Wang, Jiarui Li, Hongdan Fu

**Affiliations:** 1 College of Horticulture, Shenyang Agricultural University, Shenyang, China; 2 The Modern Facilities Horticultural Engineering Technology Center, Shenyang Agricultural University, Shenyang, China; 3 College of Agricultural Science and Technology, Shandong Agriculture and Engineering University, Jinan, Shandong, China; 4 College of Land and Environment, Shenyang Agricultural University, Shenyang, China

**Keywords:** continuous cropping, long-term organic material addition, organic carbon fraction, soil enzyme activity, tomato

## Abstract

In recent years, there has been increasing emphasis on the sustainable organic material reutilization, including straw and biochar. This study investigates the effects of prolonged applications of straw (R), biochar (B), and their combination (RB) on soil organic carbon fractions, enzyme activities, and tomato yield under continuous greenhouse cultivation, compared to a control (CK). The results showed that organic amendments reduced soil bulk density. Compared to the CK treatment, the R treatment caused a slight decrease in soil pH, whereas the B and RB treatments significantly increased soil pH by 4.10% and 6.10%, respectively. Relative to the CK treatment, the R, B, and RB treatments significantly elevated the levels of dissolved organic carbon (DOC), easily oxidizable organic carbon (ROC), microbial biomass carbon (MBC), and particulate organic carbon (POC) by ranges of 10.28%-24.34%, 38.94%-49.69%, 109.73%-121.21%, and 102.56%-196.93%, respectively. The activities of catalase (CAT), α-glucosidase (AG), β-glucosidase (BG), β-xylosidase (XYL) and cellulase (CL) were significantly enhanced after organic material addition. Additionally, the R, B, and RB treatments increased yields by 22.61%, 18.25%, and 23.74%, respectively. By integrating long-term field observations with structural equation modeling, this study offers novel mechanistic evidence that particulate organic carbon (POC) serves as a central mediator linking organic amendments to yield improvement-a regulatory pathway that has not been systematically elucidated in previous research, offering a mechanistic basis for sustainable soil management in continuous greenhouse production.

## 1 Introduction

China’s substantial population and significant demand for agricultural products have prompted farmers to adopt monoculture practices to enhance efficiency. Nevertheless, the prolonged practice of continuous cropping has resulted in soil degradation, a phenomenon that exacerbates with the increasing number of crop cycles and the presence of stubble, particularly in facility-based cultivation systems (Beretta-Blanco et al., 2019; Zhou et al., 2021). Research indicates that the disorder associated with continuous cropping negatively impacts the yield quality of various crops, including Chinese herbs and peanuts, as well as the growth of fruits and vegetables, ultimately leading to a deterioration in product quality over time (Liu et al., 2019; Han et al., 2022; Li et al., 2023).

As a foundational component of terrestrial carbon pools, SOC content exerts a direct impact on three key soil-plant-microbe processes: crop nutrient uptake capacity, root growth dynamics, and soil microbial community activity (Ge et al., 2014; Pan et al., 2025). Soil organic carbon (SOC) comprises both biogenic and fossil-derived fractions, which are influenced by soil management practices (Galy et al., 2015; Liu et al., 2026). Continuous cropping has been shown to adversely affect SOC dynamics (Allam et al., 2022; Zhang et al., 2025). The advantages associated with the enhancement of soil organic matter through continuous cropping are primarily of a short-term nature (Dou et al., 2016; Angst et al., 2024). For instance, the practice of continuous rice cropping has been shown to diminish the quantity of SOC and the rates of mineralization, while simultaneously increasing the light fraction and particulate organic matter (Singer et al., 2019; Li et al., 2025b). Furthermore, the chemical composition of SOC undergoes alterations, characterized by a reduction in aromatic carbon and a decline in soil humidification within the 0–20 cm soil layer (Zhou et al., 2020). However, continuous cropping is associated with a decrease in both active and stable organic carbon levels. Active organic carbon, which decomposes rapidly, serves as an indicator of early changes, whereas stable organic carbon exhibits greater persistence within the soil matrix (Li et al., 2021, 2022). Taken together, these findings indicate that different organic carbon fractions are influenced by crop types and various continuous cropping environments under different cultivation conditions. This has further increased the uncertainty in our understanding of their changes.

Organic materials derived from plant and animal sources are abundant in carbon and nitrogen, providing prolonged fertilization benefits and are readily accessible (Zhang et al., 2019). Their use improves soil structure and nutrient availability, thereby promoting crop growth and supporting sustainable ecological agricultural practices (Su et al., 2004; Zhong et al., 2022; Edussuriya et al., 2023; Ling et al., 2025). Amending soil with organic materials necessitates the application of substantial quantities to enhance conditions for successive cropping cycles. This approach not only improves the physical structure of the soil but also alters its chemical properties and stimulates microbial activity. The incorporation of diverse organic materials significantly affects the soil’s physicochemical characteristics, microbial communities, and overall crop productivity (Ye et al., 2018; Duan et al., 2025). Furthermore, the introduction of organic materials elevates the content of soil organic matter, thereby enhancing the soil carbon pool (Paustian et al., 2016). Returning straw to the field enriches the soil with organic matter and essential nutrients, facilitating the proliferation of beneficial microbial phyla such as Acidobacterium and Anabaena. Additionally, long-term straw incorporation enhances enzyme activity and microbial biomass carbon, which in turn promotes organic carbon mineralization and supplies vital nutrients for plant growth (Verchot and Borelli, 2005; Yuan et al., 2024; Li et al., 2025a). Straw addition also elevates cellulose and hemicellulose levels, thereby increasing the activities of enzymes such as β-glucosidase, cellobiose hydrolase, and β-xylosidase (Wu et al., 2024). This practice not only mitigates air pollution associated with straw burning but also improves soil fertility and contributes to increased crop yields (Triberti et al., 2008; Li et al., 2021; Yang et al., 2023). Moreover, biochar, characterized by an alkaline pH ranging from approximately 7 to 10, serves as an effective agent for mitigating soil acidity (Chan et al., 2007; Zhang et al., 2022; Pan et al., 2025). Empirical studies indicate that biochar enhances the soil’s capacity to retain water and nutrients while simultaneously reducing emissions of nitrous oxide (N_2_O), carbon dioxide (CO_2_), and methane (CH_4_) (Thomazini et al., 2015; Woolf et al., 2016). Additionally, it promotes the release of soluble carbon and micronutrients (Woolf et al., 2016). Soil enzymes, produced by microorganisms, plants, and animals, serve as indicators of soil fertility (Fukumasu and Shaw, 2017). Biochar has been shown to modulate the activities of enzymes such as β-glucosidase, arylsulfatase, and urease; however, it does not significantly enhance the soil fungal community (Lopes et al., 2021; Zhu et al., 2021). Under greenhouse intensive conditions, the response patterns of enzymes to the addition of different organic materials and their relative contributions to yield changes remain unclear.

Tomato is China’s largest protected vegetable crop, but intensified cultivation has exacerbated continuous cropping obstacles, such as acidification, pests and diseases, which restricting industrial sustainability (Parastesh et al., 2019; Chen et al., 2021; Wang et al., 2022, 2026). Organic materials are commonly used for mitigation, improving soil organic matter while enhancing the economic benefits of tomatoes. We hypothesize that the combined application of straw and biochar will synergistically improve soil organic carbon fractions, enzyme activities, and tomato yield more effectively than either amendment alone. This improvement is expected to result from enhanced soil physicochemical properties, stimulated microbial activity, and increased accumulation of soil organic carbon, with labile organic carbon fractions playing a key mediating role linking soil properties to tomato yield under continuous greenhouse cultivation. While previous studies have primarily focused on short-term effects or single amendments, long-term comparative studies examining the differential impacts of straw, biochar, and their combination on soil carbon fractions and enzyme activities remain scarce. Furthermore, the mechanistic pathways through which these amendments influence crop yield remain largely unexplored. Therefore, we established four treatments with different organic material additions. We analyzed soil and plant characteristics, evaluated enzyme activities and functional traits, with three main objectives: (i) to investigate the response of soil enzyme activity to organic amendments; (ii) to assess how these enzymes affect different organic carbon fractions; and (iii) to apply structural equation modeling (SEM) to clarify how organic material additions influence tomato yield.

## 2 Materials and methods

### 2.1 Experimental site

This experiment was carried out in greenhouse (41^°^48’N, 123^°^25’E) of Shenyang Agricultural University. The original soil was taken from the masan Commune of Ramatai Village, Yuhong District, Shenyang City, Liaoning Province, which has been growing tomatoes twice a year since 1990. The soil type is brown soil. The chemical properties of the cultivated soil layer (0–20 cm) are shown in **Supplementary Table 1**. During the experiment, the greenhouse was maintained at an average temperature of 25 ± 5°C during the day and 18 ± 3°C at night, with relative humidity ranging from 65% to 75%. Natural light was supplemented with artificial lighting to ensure a 14-hour photoperiod.

### 2.2 Experimental design

The experiment was conducted in an experimental greenhouse equipped with multiple cement cultivation pools, each measuring 1.5 m in length, 1 m in width, and 0.8 m in depth (Supplementary **Figure 1**). Prior to the spring cropping season of 2009, each cultivation pool was filled with *in-situ* original soil. Commencing with the 2009 spring crop, a long-term soil organic material addition and restoration experiment was implemented in a double-cropping greenhouse system over a one-year annual rotation cycle. A completely randomized block design was employed for the experiment, with three replications per treatment to ensure statistical reliability.

**Figure 1:**
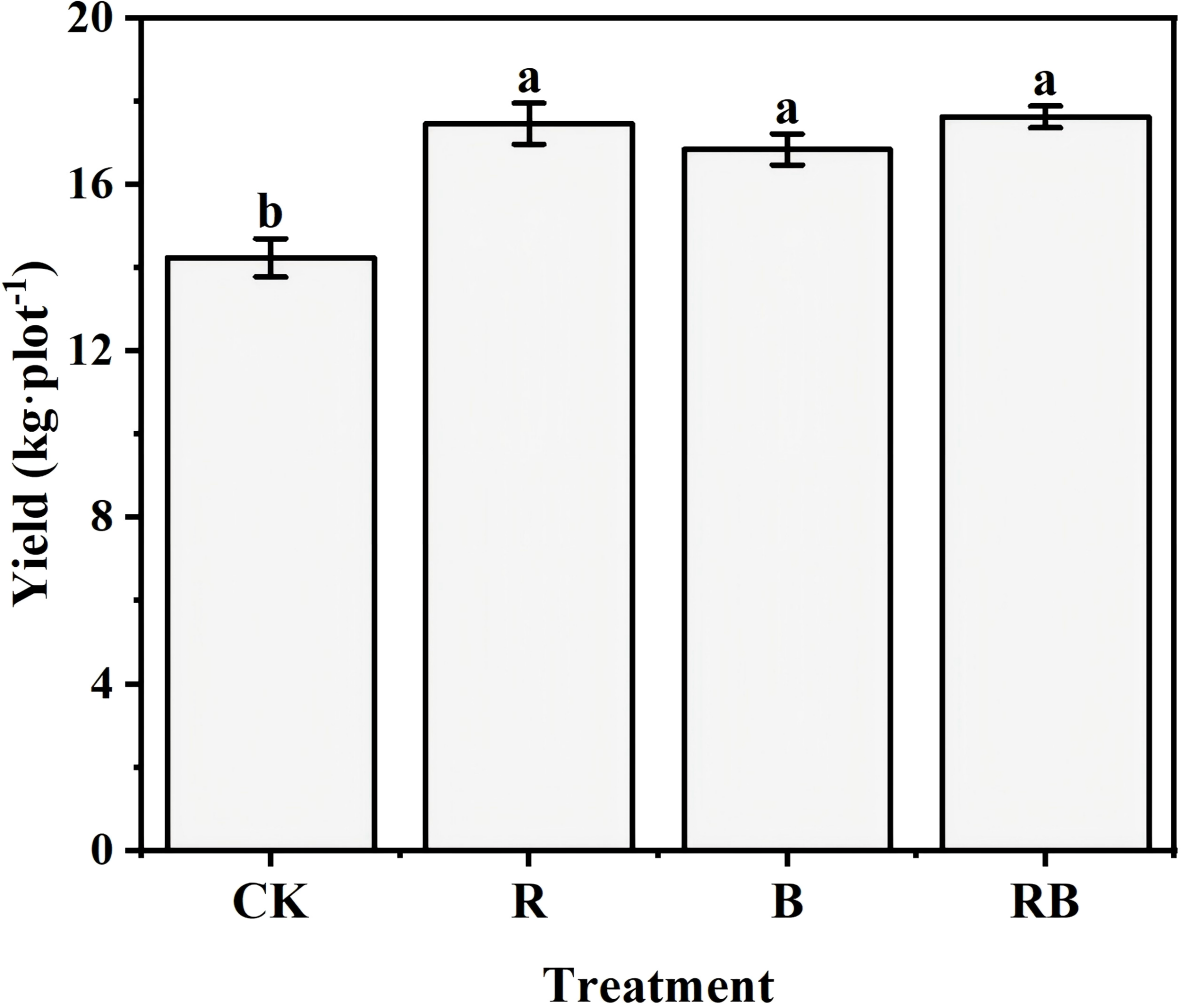
Long-term organic amendments impact addition on tomato yield under continuous tomato cropping. Note: Error bars represent standard error.CK, control (no material addition), R, straw; B, biochar; RB, straw+biochar. Different lower-case letters indicate significant differences between samples (n=3*, p*< 0.05).

Four experimental treatments were established in this study: a control treatment with no organic material addition (CK), maize straw addition (R), biochar addition (B), and combined maize straw and biochar addition (RB). The test organic materials were prepared as follows: maize (Zea mays L.) straw was sun-dried to a constant weight and crushed to a particle size of 2–3 cm, with further processing to pass through a 5 mm sieve; the crushed straw exhibited a carbon-to-nitrogen (C/N) ratio of 65.3, with 78% of particles ranging from 2 to 5 mm and 22% of particles smaller than 2 mm. The test biochar was derived from corn cobs via slow pyrolysis under oxygen-limited conditions: specifically, corn cobs were carbonized in a vertical tubular furnace (Model GSL-1700X, MTI Corporation, Richmond, CA, USA) at 500°C for 3 hours, with a heating rate of 10°C min^-^¹ and a 3-hour residence time at the target temperature. Nutrient contents of the basal fertilizer and organic amendments are detailed in **Supplementary Table 2**. All treatments received 4.50 kg plot^-^¹ of well-decomposed chicken manure as the basal fertilizer, which was uniformly incorporated into the 0–20 cm plow layer prior to tomato transplanting. For the organic material addition treatments, 2.10 kg plot^-^¹ of maize straw was applied in the R treatment, 1.10 kg plot^-^¹ of biochar was applied in the B treatment, and both 2.10 kg plot^-^¹ of maize straw and 1.10 kg plot^-^¹ of biochar were applied in the RB treatment. To mitigate nutrient imbalances induced by the incorporation of maize straw and biochar, fertilization rates were adjusted based on the nutrient content analysis of the organic amendments, in combination with nutrient release patterns determined via preliminary incubation experiments (**Supplementary Table 3**). Compared with the CK treatment, nitrogen fertilizer application rates were reduced by 32%, 18%, and 49% in the R, B, and RB treatments, respectively, to offset the nitrogen released during straw decomposition. Correspondingly, phosphorus and potassium fertilizer application rates were also optimized in line with the nutrient supply characteristics of each organic amendment.Topdressing was implemented at two critical growth stages: the first topdressing was applied during the expansion stage of the first fruit on the first tomato cluster, and the second topdressing was administered during the expansion stage of the second fruit. The long-term field experiment was initiated in the spring of 2009, and soil amendments have been consistently applied during each tomato cropping season thereafter. This study focuses on data collected from the 2022 autumn tomato cropping season, marking the 14th consecutive year of the experimental treatments.

The tomato cultivar utilized in the experiment was Jin Wo No. 6886, provided by the Shenyang Academy of Agricultural Sciences, Liaoning Province, China. Tomato seedlings were transplanted on August 29, 2022, and fruits were harvested at physiological maturity upon the development of four fruit clusters. Both soil and plant samples were systematically collected from the 2022 autumn tomato cultivation plots for subsequent laboratory analyses and index determination.

### 2.3 Soil and fruit sampling

Tomato yield was determined by harvesting all ripe fruits from each plot at four stages corresponding to the maturity of each fruit cluster. Fruits were counted and weighed using an electronic balance (accuracy ± 0.1g), and total yield per plot was calculated by summing the yields from all four harvests. Yield was expressed as kg per plot, with each plot area being 1.5 m². When the tomato was harvested in late December 2022, five-point samples were taken from a depth of 20 cm in each cultivation pond, and the soil was thoroughly mixed through a 2 mm sieve to remove stones and coarse roots from the soil. These soil samples were divided into two parts: one part was naturally air-dried in a dry and cool place for the determination of the basic physical and chemical properties of the soil as well as the organic carbon fractions; the other part of the fresh soil samples was stored at temperatures 4°C to determine the soil microbial biomass carbon and soil enzyme activity.

### 2.4 Determination of soil physiochemical property

For soil physiochemical analysis, refer to Wang et al. (2025b). The bulk density of the soil is determined via the ring sampler method. After the samples were naturally air dried to a constant quality, they were placed on the machine again and weighed. The soil was filtered after 30 min of shaking at room temperature, the pH was measured with a pH meter at a water–soil ratio = 2.5:1, and the conductivity was measured with a conductivity meter at a water–soil ratio = 5:1. Total organic carbon (TOC)/total nitrogen (TN) analysis was used to determine the soil total nitrogen and organic carbon contents (multi N/C 3100, Analytikjena, Germany) (Bond-Lamberty et al., 2016). The total phosphorus (TP) content was determined via the molybdenum-antimony anti-colorimetric method after digestion. The total potassium (TK) was determined by a flame photometer with unused digestion solution (Nobile et al., 2020). The available nitrogen (AN) of the soil was determined via the alkaline hydrolysis diffusion method. The soil available phosphorus (AP) was determined via the NaHCO3 extraction-molybdenum-antimony method. The available potassium of (AK) the soil was extracted with NH4OAc and determined with a flame photometer (Wang et al., 2025b).

### 2.5 Determination of soil organic carbon fraction

TOC concentration measured in the it’s not fumigated soil extract was used to determine the dissolved organic carbon (DOC) content (Chen et al., 2021). The content of easily oxidizable organic carbon (ROC) was determined via the potassium permanganate oxidation method 25 ml of 333 mmol L^-1^ potassium permanganate was added to the soil, and then colorimetric analysis was performed at 565 nm with a spectrophotometer after shaking and centrifugation (Blair et al., 1995). Soil particulate organic carbon (POC) was determined using the method described by German et al (German et al., 2011). Briefly, 20 g of soil was passed through a 2 mm sieve, and 100 mL of a 5 g L^-1^ sodium hexametaphosphate solution was added. The mixture was continuously shaken at 90 rpm for 18 hours at room temperature, then passed through a 53 μm sieve and rinsed repeatedly with deionized water. The residual particles on the sieve were collected, dried at 40°C, and weighed (Ndzana et al., 2022). The mineral-associated organic carbon (MAOC) content was calculated as the difference between the total organic carbon and particulate organic carbon (Elliott and Coleman, 1992). Microbial biomass carbon (MBC) content was determined via fumigation extraction with 0.5 mol L^-1^ K_2_SO_4_ at a water–soil ratio of 4:1. The samples were then centrifuged at 4000 rpm for 5 minutes, and the filtrate was passed through a 0.45 μm filter membrane for determination via a TOC/TN analyzer:


$$MBC=(C_{fumigated}-C_{non-fumigated})/K_{EC}$$

where Cfumigated represents the organic carbon concentration in the extract of fumigated soil. Cnon-fumigeted represents the organic carbon concentration in the extract of non-fumigated soil samples. KEC represents the extractable proportion of carbon released by microbes after fumigation, the commonly used value is 0.45.

### 2.6 Determination of soil enzyme activity

The soil enzyme activities of soil α-glucosidase (AG, EC 3.2.1.20), β-glucosidase (BG, EC 3.2.1.21), β-xylosidase (XYL, EC 3.2.1.37) and N-acetyl-β-D-glucosaminidase (NAG, EC 3.2.1.52) were determined via a nitrophenol colorimetric assay (Verchot and Borelli, 2005). The activity of catalase (CAT, EC 1.11.1.6) was determined via potassium permanganate titration (Wang and Sheng, 2023). The activity of cellulase (CL, EC 3.2.1.4) was determined via the 3,5-dinitrosalicylic acid (DNS) colorimetric method (Sharma et al., 2016).

### 2.7 Statistical analysis

After the original experimental data were processed in Excel, SPSS Statistics 19.0 (IBM, New York, USA) was used for one-way analysis of variance (ANOVA). Duncan’s test was employed and the difference of p< 0.05 was considered significant. Origin 2022 was used to plot the above analysis results. The variations in soil organic carbon fractions and enzyme activities among four treatments were investigated using the principal component analysis (PCA) combined with ANOSIM test. The relationships among the enzyme activities, organic carbon fractions and soil physiochemical properties were determined via the Mantel test. Redundancy analysis (RDA) was used to explore the relationships between enzyme activity and organic carbon fractions. Structural equation modelling (SEM) was used to explore the causal relationships among the soil properties, enzyme activities, organic carbon fraction and tomato yield via AMOS 21.0 (SPSS Inc., Chicago, IL, USA).

## 3 Results

### 3.1 Long-term organic amendments impact soil properties and tomato yield

Compared to the control (CK), the addition of various materials to the soil led to a notable reduction in soil bulk density, with the most pronounced decrease occurring in the B treatment (**Table 1**). The soil pH levels in both the B and RB treatments were significantly higher than those in the control, exhibiting increases of 4.10% and 6.10%, respectively. Conversely, a decline in soil pH was observed in the R treatment. The soil electrical conductivity (EC) value experienced a significant increase under the B treatment, with the most substantial rise recorded at 45%. The concentrations of total nitrogen, total phosphorus, total potassium, available phosphorus, and available potassium were significantly elevated compared to the CK treatment, with increases ranging from 24% - 44%, 9.46% - 20%, 7.78% - 21%, 18% - 27%, and 19% - 24%, respectively. Additionally, the available nitrogen content in the R treatment showed a significant increase of 17.35% relative to the CK treatment. Moreover, the incorporation of organic materials significantly improved tomato yield. however, no significant differences were detected among the three treatments (**Figure 1**).

**Table 1:** Long-term organic amendments impact soil physicochemical properties of continuously cropped tomato.

Physicochemical property	CK	R	B	RB
pH	6.99 ± 0.09bc	6.85 ± 0.17c	7.28 ± 0.14ab	7.42 ± 0.04a
BD (g cm^-3^)	0.91 ± 0.01a	0.64 ± 0.04c	0.79 ± 0.04b	0.63 ± 0.01c
EC (μs cm^-1^)	985 ± 43b	1227 ± 30ab	1424 ± 234a	1332 ± 24ab
TN (g kg^-1^)	3.44 ± 0.03c	4.35 ± 0.06b	4.27 ± 0.17b	4.94 ± 0.10a
TP (g kg^-1^)	8.98 ± 0.10c	10.22 ± 0.13ab	9.83 ± 0.30bc	10.76 ± 0.42a
TK (g kg^-1^)	15.56 ± 0.41c	16.77 ± 0.17b	17.18 ± 0.23b	18.89 ± 0.24a
AN (mg kg^-1^)	214 ± 5.55b	251 ± 10.43a	226 ± 3.32ab	235 ± 16.17ab
AP (mg kg^-1^)	247 ± 7.05b	315 ± 5.05a	293 ± 9.86a	314 ± 10.15a
AK (mg kg^-1^)	569 ± 17.32b	706 ± 11.61a	679 ± 9.74a	705 ± 14.42a

### 3.2 Long-term organic amendments impact on soil organic carbon in tomato continuous cropping

**Figure 2:**
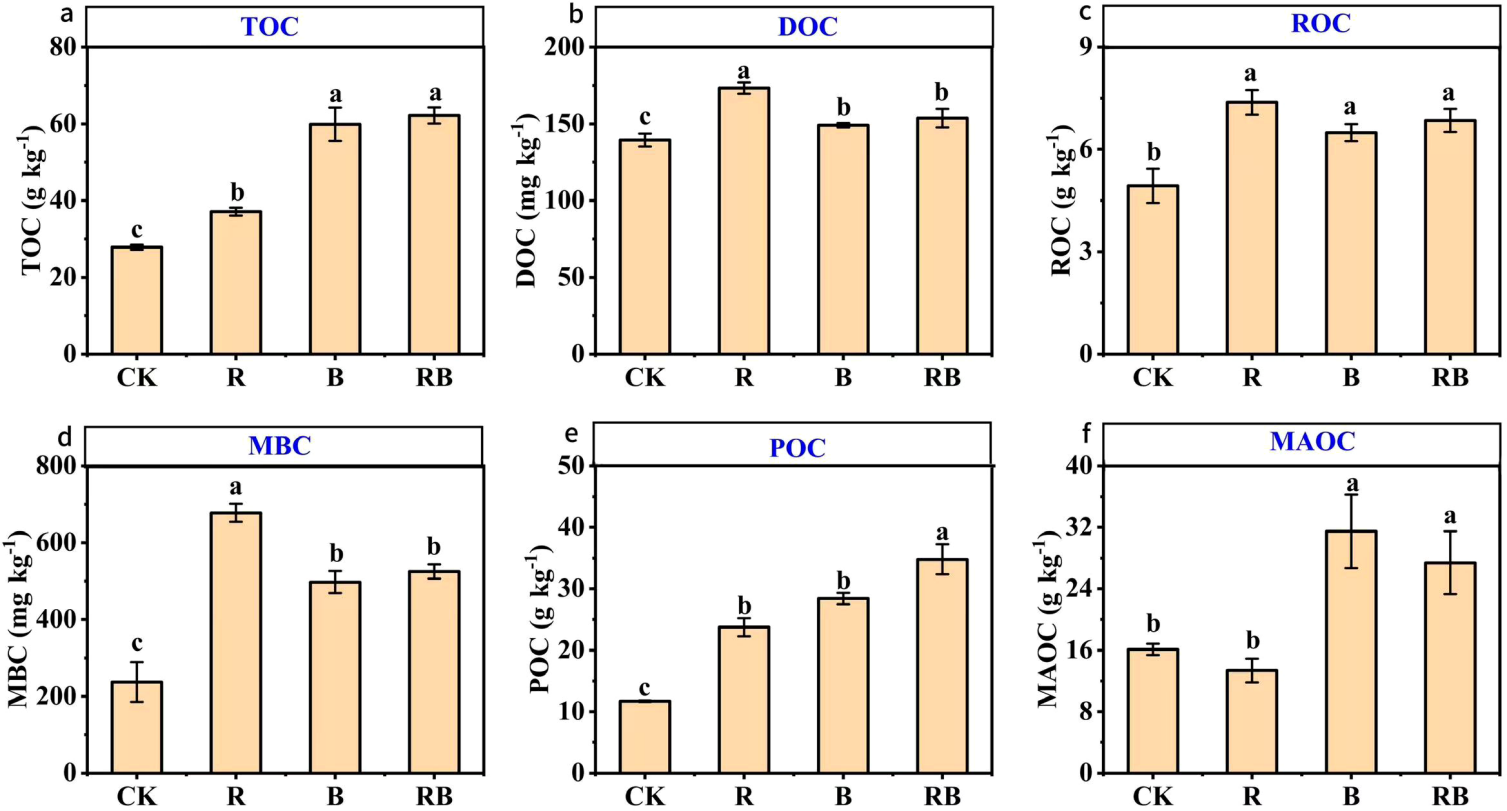
Effects of long-term organic amendments on organic carbon fractions in tomato continuous cropping. Note: Error bars represent standard error. CK, control (no material addition), R, straw; B, biochar; RB, straw+biochar. Different lower-case letters indicate significant differences between samples (n=3,*p*< 0.05). **(a)**:TOC; **(b)**:DOC; **(c)**:ROC; **(d)**:MBC; **(e)**:POC; **(f)**:MAOC.

Compared to the control (CK), the addition of organic materials significantly increased the total organic carbon content, with the following order observed: RB > B > R > CK (**Figure 2**). The incorporation of these materials notably enhanced the soil’s labile organic carbon levels. The concentrations of soil DOC, ROC, and MBC were highest in the R treatment. In comparison to the CK, the concentration of soil particulate organic carbon (POC) increased significantly by 103% - 197%. The content of mineral - associated organic carbon (MAOC) varied to different extents following the addition of various materials, with the lowest value recorded in the R treatment at 13.36 g kg-1, which was significantly lower than that in the B and RB treatments.

**Figure 3:**
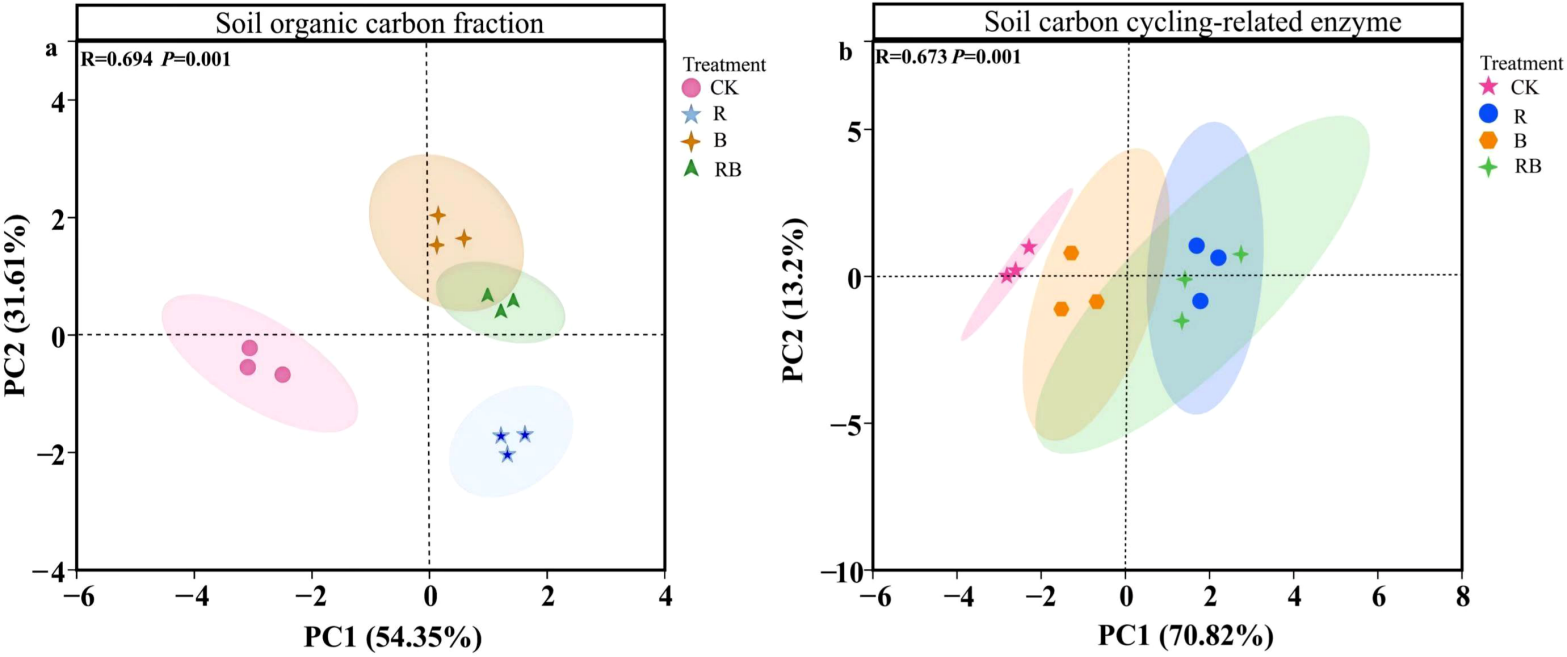
Principal component analysis of organic carbon fractions **(a)** and carbon cycling-related enzyme activities **(b)** in tomato continuous cropping soil by long-term organic improvement.

Principal component analysis (PCA) of organic carbon fractions in tomato continuous cropping soil by long-term organic amendments (**Figure 3a**). The PCA results showed that the CK treatment was well separated from the other three treatments along the PCA1 axis, and the two coordinate axes together explained 86% of the variance. The ANOSIM results further confirmed that continuous cropping had a significant impact on soil organic carbon fractions (R²=0.694, *P* = 0.001).

### 3.3 Long-term organic amendments impact on soil carbon cycling-related enzyme activities under continuous tomato cropping

**Figure 4:**
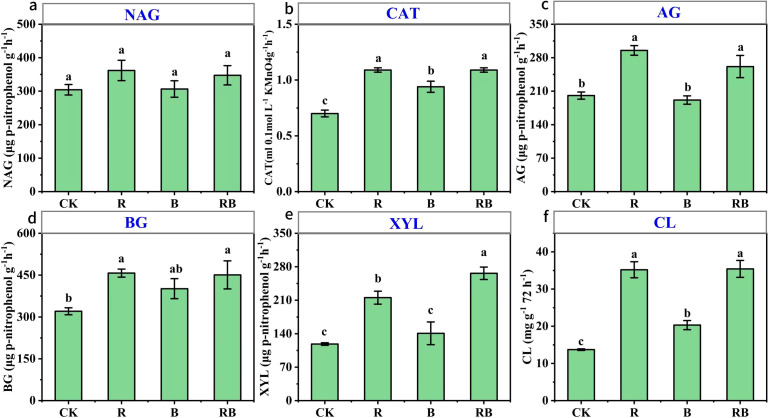
Long-term organic amendments impact on soil carbon cycling-related enzyme activities under continuous tomato cropping. Note Error bars represent standard error. CK, control (no material addition), R, straw; B, biochar; RB, straw+biochar. Different lower-case letters indicate significant differences between samples (n=3, *p*< 0.05). **(a)**:NAG; **(b)**:CAT; **(c)**:AG; **(d)**:BG; **(e)**:XYL; **(f)**:CL.

The activity of catalase was significantly greater than that of CK across all treatments, with CAT activity peaking under the RB treatment, showing an increase of 57%. The intensity of α-glucosidase activity followed the order R > RB > CK > B, with the highest activity recorded in continuously cropped tomato soil with straw (295 μg p-nitrophenol g^-1^ h^-1^). Compared with that in the CK treatment, the activity of β-glucosidase in the R and RB treatments increased by 41% and 43%, respectively. Following the addition of straw, the activity of β-xylosidase was significantly higher than that observed in the CK and biochar treatments. Additionally, soil cellulase activity in the other three treatments showed a significant increase compared to the CK treatment. However, no significant changes in N-acetyl-β-D-glucosaminidase activity were observed across all treatments. (**Figure 4**).

Principal Component Analyses (PCA) of carbon cycle enzymes in tomato continuous cropping soil under long-term organic amendments (**Figure 3b**). The PCA results showed that the CK treatment was well separated from the other three treatments along the PCA1 axis, and the two coordinate axes together explained 84% of the variance. The ANOSIM results further confirmed that continuous cropping had a significant impact on soil carbon cycle enzymes (R²=0.673, *P* = 0.001).

### 3.4 Relationships between soil physiochemical properties, enzyme activities and organic carbon fractions

**Figure 5:**
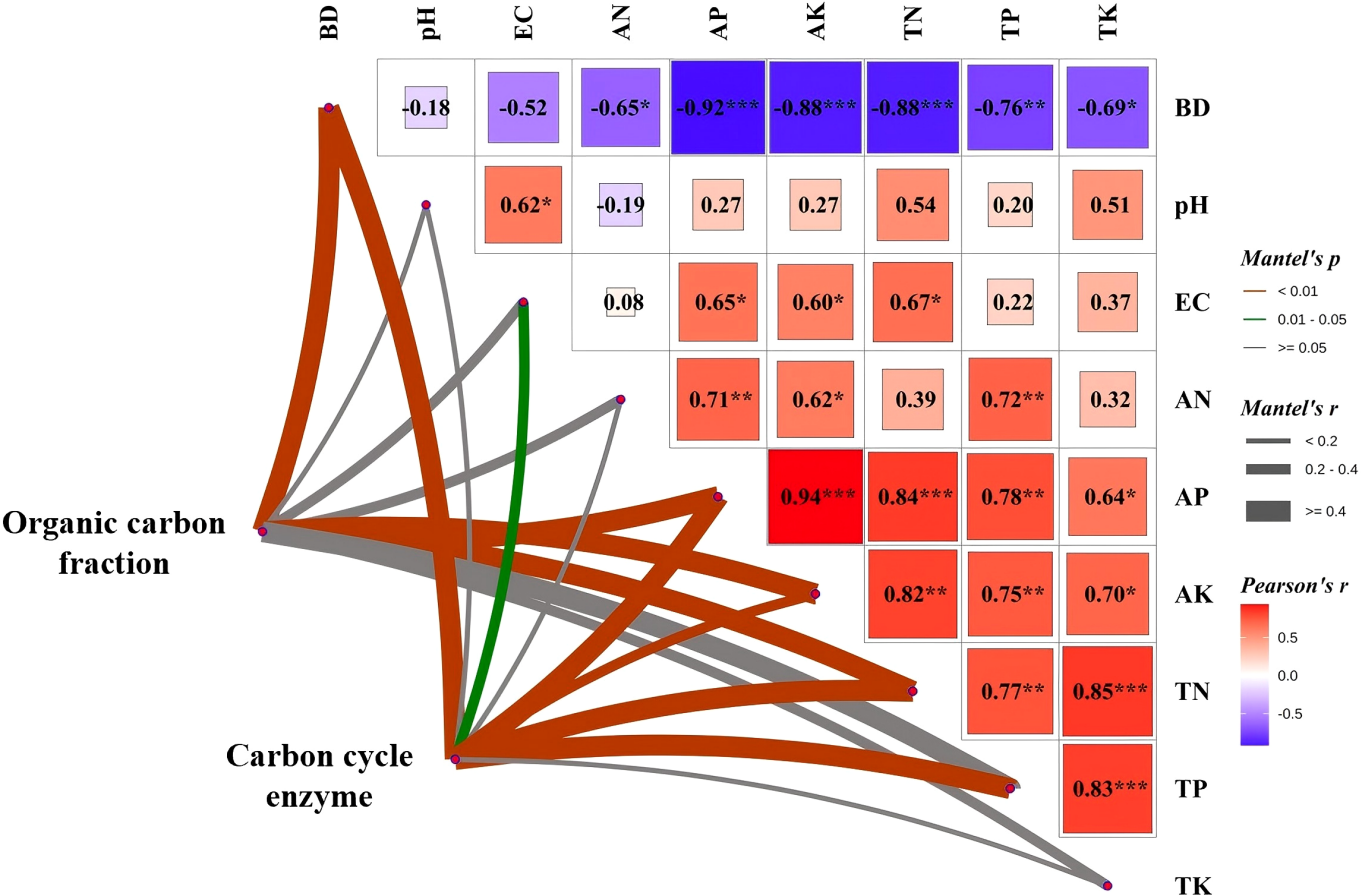
The soil organic carbon fraction and enzyme activity were linked to each factor via Mantel tests under long-term material addition for continuous tomato cropping (*** *p<* 0.001, ** *p*< 0.01,*p<0.05).

**Figure 6:**
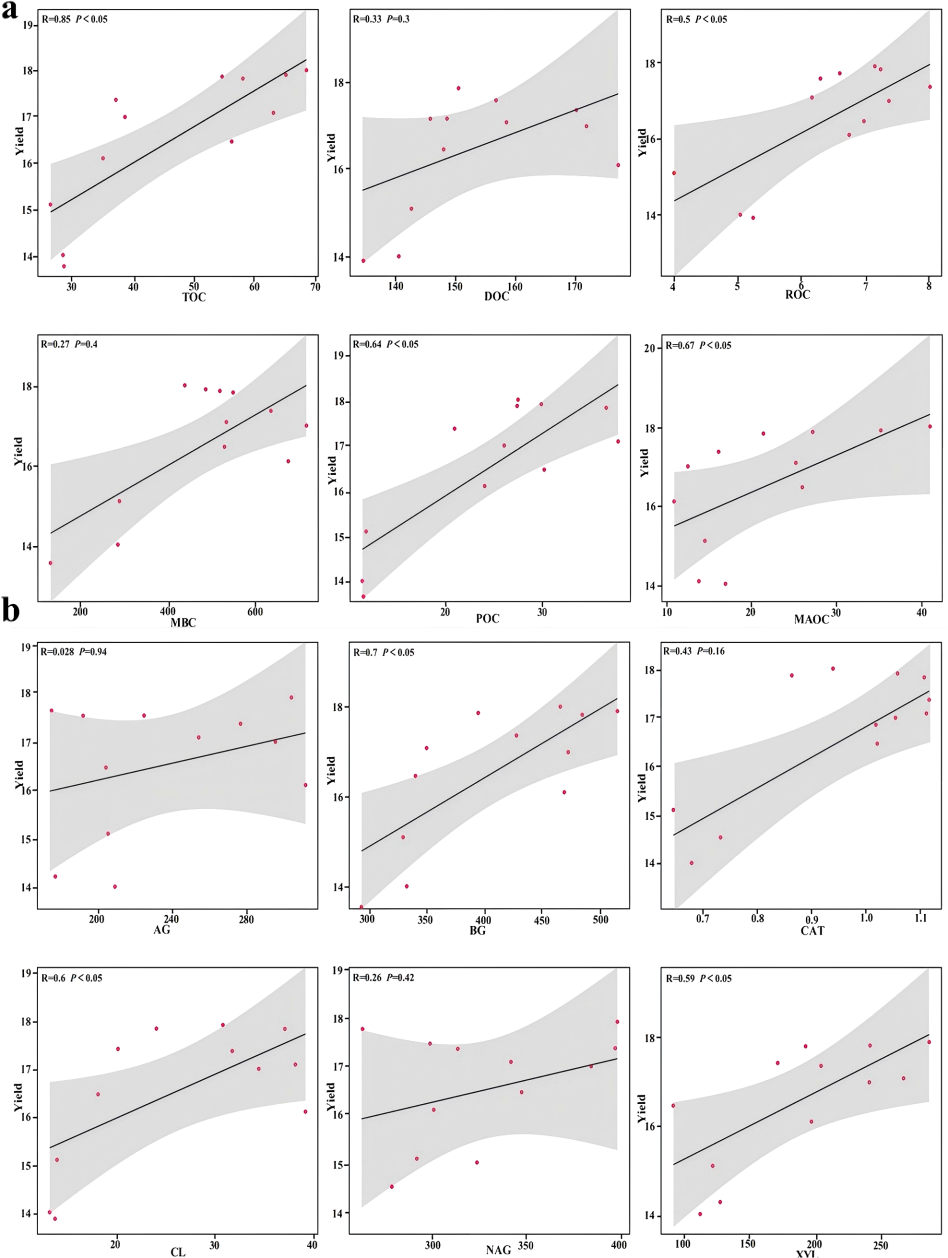
Correlation analysis between soil organic carbon fractions **(a)**, activities of enzymes related to the soil carbon cycle **(b)** and tomato yield.

**Figure 7:**
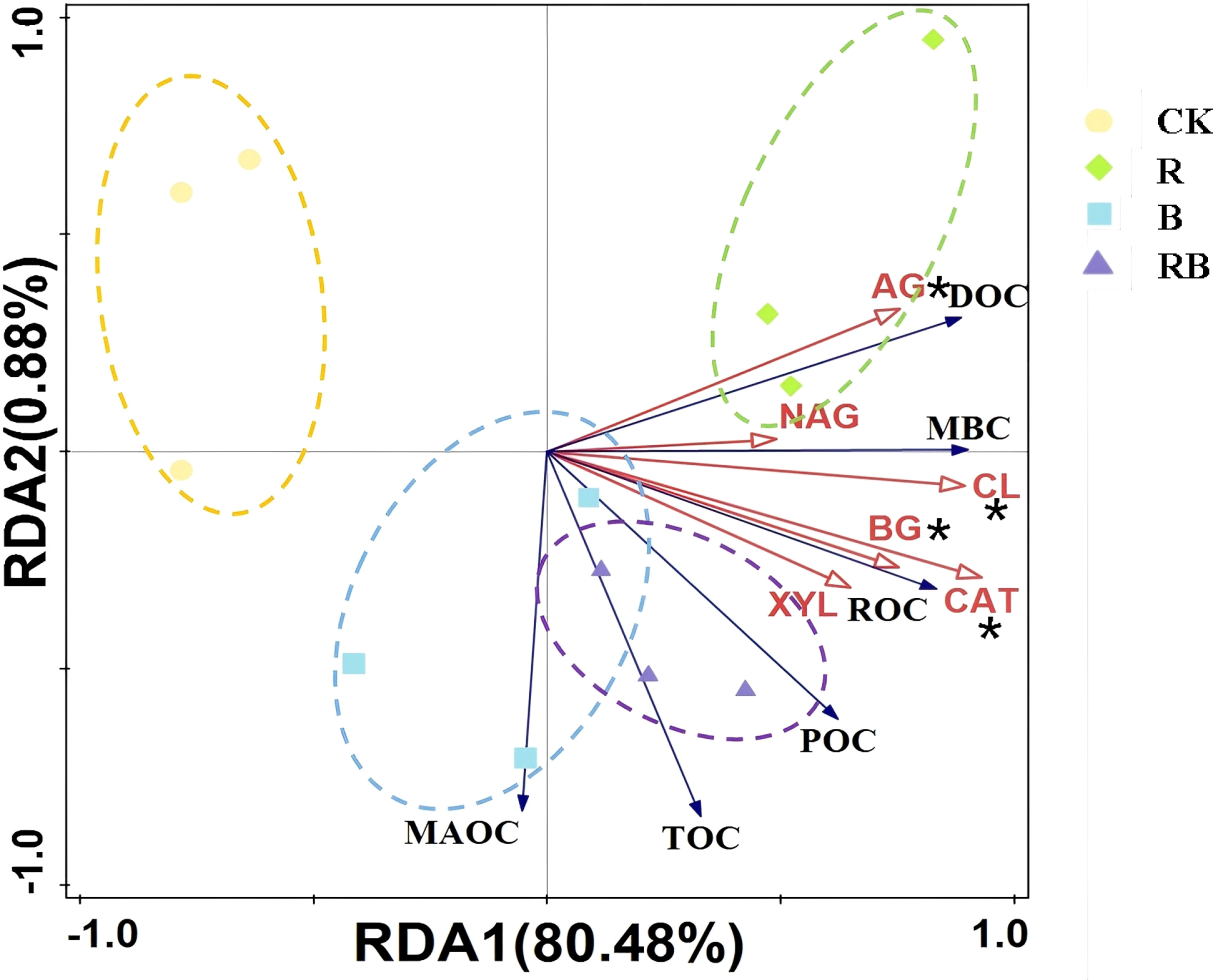
RDA of the soil organic carbon fraction and soil enzymes associated with carbon cycle under long-term material addition for continuous tomato cropping. (*p< 0.05).

Mantel text showed that the soil organic carbon fractions were significantly correlated with BD, AP, AK, TN and TP, and the enzyme activities were significantly correlated with BD, EC, AP, AK, TN and TP (**Figure 5**). The results revealed a significant positive correlation between soil organic carbon fractions (TOC, ROC, POC, MAOC) and soil carbon cycle enzyme activity (BG, CL, XYL) with tomato yield based on Pearson correlation analysis (**Figure 6**). RDA revealed that soil enzymes explained 81% of the total changes in organic carbon fractions, with CAT, CL, AG and BG having a significant impact on organic carbon fractions (**Figure 7**).

## 4 Discussion

### 4.1 Organic material addition can effectively improve soil physicochemical properties

Returning straw to the field can improve soil bulk density and porosity, enhance physical properties, and increase the capacity for soil to supply water, fertilizer, gas, and heat, thereby boosting crop yields (Dong et al., 2018; Urra et al., 2018; Chen et al., 2023). The experiment demonstrates that the bulk density of soil treated with various materials was significantly lower than that of the control group, with straw application (R and RB treatments) proving the most effective. This effect may be attributed to the fact that straw application promotes the expansion of tomato roots and contributes to soil aeration (Yang et al., 2023; Liu et al., 2024). Biochar is alkaline, contains abundant mineral nutrients, and has a porous structure. When applied to soil, it can significantly increase soil pH, creating an optimized root environment that promotes crop growth, which aligns with our observations in the B treatment (Wang et al., 2021a; Chen et al., 2023; Yuan et al., 2024). Following the addition of organic materials, the nutrient content of each treatment was greater than that of the control. The total nitrogen, total phosphorus, available nitrogen, and available potassium contents of the soil all improved significantly. Notably, the addition of straw increased the input of carbon substrates into the soil, which can stimulate microbial growth and increase microbial biomass. According to ecological stoichiometry theory, the rapid expansion of microbial populations elevates their demand for nutrients such as phosphorus, potentially leading to microbial P limitation. Under such conditions, microorganisms may invest energy in producing extracellular enzymes (e.g., phosphatases) and organic acids to mineralize organic phosphorus or solubilize insoluble phosphorus compounds, thereby increasing the concentration of bioavailable phosphorus in the soil (Wang et al., 2021a; Pang et al., 2024; Yuan et al., 2024). Additionally, due to the unique pore structure and high cation exchange capacity of biochar, its addition enhances soil nutrient availability and helps maintain a better balance of nutrients in the soil (Wang et al., 2021b). As a result, tomatoes can absorb more available potassium from the soil, which supports fruit development and quality regulation.

The maize straw used in this study had a high C:N ratio (65.3), which is generally expected to induce strong microbial nitrogen immobilization during the early stage of decomposition, often resulting in temporary nitrogen limitation for crops in short-term studies (Kuzyakov and Xu, 2013). However, in the present study, chemical nitrogen fertilizer inputs were reduced by 32%, 18%, and 49% in the R, B, and RB treatments, respectively, yet both soil nitrogen availability and tomato yield increased compared with the control. This contrasting result may be attributed to the long-term nature of the experiment. After 14 years of continuous organic material application, the soil system may have gradually reached a new dynamic equilibrium of nutrient cycling. Long-term straw incorporation likely promoted the accumulation of soil organic matter and enhanced microbial activity (Yan et al., 2007), allowing continuous nitrogen mineralization from the accumulated organic pool to offset the initial microbial immobilization associated with high C:N residues (Li et al., 2026).Therefore, the system may shift from short-term plant–microbe nitrogen competition toward a more stable plant-microbe nutrient synergy, highlighting the importance of long-term experiments in understanding soil nutrient dynamics under organic residue management.

### 4.2 Changes in soil organic carbon fractions after the addition of organic materials

**Figure 8:**
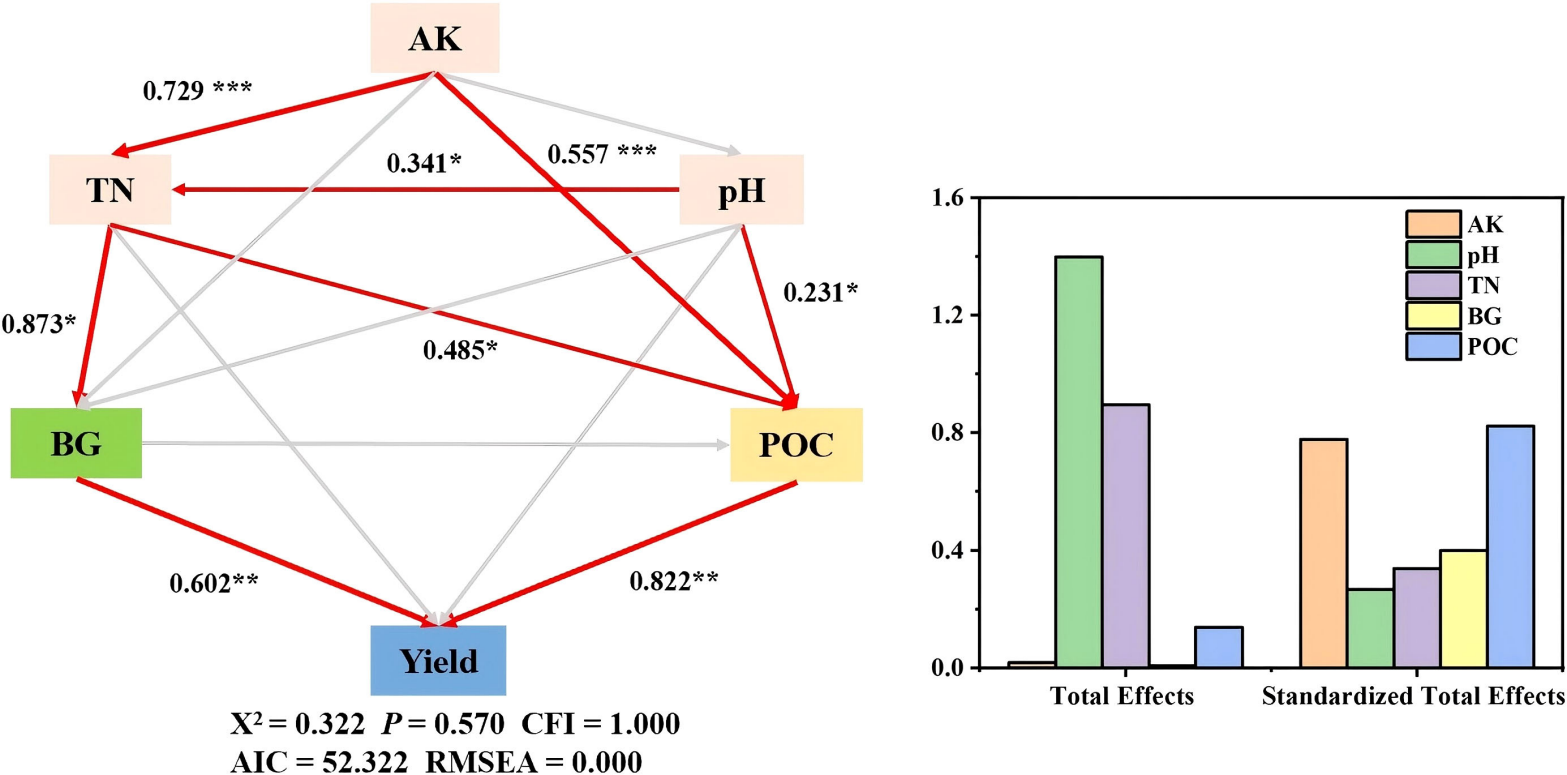
The structural equation model (SEM) shows the potential causal relationships among soil physicochemical properties (AK, TN, pH), enzyme (BG), the organic carbon fraction (POC) and tomato yield after the addition of organic material. Red and gray arrows represent significant positive and insignificant pathways, respectively. R2 near the observed parameters denotes the proportion of the variance explained by other variables in the model (***p< 0.001, **p< 0.01, *p< 0.05).

Soil organic carbon is crucial for preserving soil structural integrity and improving the retention of water and fertilizers (Meng et al., 2023). The findings indicate that the incorporation of rice straw and biochar significantly enhanced the total organic carbon levels in soil under continuous tomato cultivation, with the highest organic carbon content observed in the B treatment and the combined RB treatment. This enhancement is likely attributable to biochar’s inherent nature as a carbon-rich organic material, which facilitates the accumulation of soil organic carbon by directly introducing external carbon sources (Li et al., 2020). Furthermore, dissolved organic carbon and readily oxidizable organic carbon are responsive to alterations in land use, making them valuable indicators for detecting early changes in soil organic carbon dynamics (Bongiorno et al., 2019; Don et al., 2023). The exclusive application of straw markedly enhanced the soil concentrations of two specific organic carbon fractions, expedited the nutrient cycling process within the soil, and diminished its stability. Notably, the microbial biomass carbon content exhibited a significant increase with the introduction of various materials, particularly under the treatment involving only rice straw. This phenomenon may be attributed to the ability of straw to effectively enhance soil structure, supply abundant carbon and nitrogen sources for microbial populations, and create favorable conditions for their growth and metabolic activities (Gupta Choudhury et al., 2014). Conversely, the microbial biomass carbon content in treatments incorporating biochar was found to be lower than that in treatments utilizing straw. This discrepancy may be explained by the elevated carbon-to-nitrogen (C/N) ratio associated with biochar application, which hinders the absorption and utilization of inert carbon by microorganisms. As a product resulting from the transformation of plant residues into soil humus, POC is particularly vulnerable to disturbances, and fluctuations in POC levels can significantly influence the stability of organic carbon within the soil, ultimately impacting plant root biomass (Ye et al., 2018; Chen et al., 2020). Compared with short-term studies focusing on total organic carbon, our 14-year trial provides robust evidence of the cumulative effects of organic amendments on soil carbon dynamics. A key novel contribution is the identification of POC as the dominant factor driving yield increases, explaining 82% of the cumulative impact by a structural equation model analysis (**Figure 8**), suggesting that the incorporation of organic materials enhances soil microbial activity and promotes the formation and stability of POC by facilitating the integration of organic carbon into macroaggregates, thereby creating favorable microhabitats for crop growth and development (Li et al., 2021). Furthermore, MAOC interacts with soil clay particles, exhibiting considerable stability. In this investigation, biochar was found to significantly augment the MAOC content, as the carbon present in biochar is predominantly highly aromatic and inert, rendering it resistant to conversion into soluble carbon through microbial decomposition once reintroduced to the soil. This characteristic is advantageous for the preservation of carbon within the soil, thereby contributing to increased soil carbon sequestration (Mishra et al., 2021; Minasny et al., 2023).

### 4.3 Organic material addition had a positive effect on soil organic carbon-related enzyme activities

Soil enzymes represent a highly dynamic organic component of soil, playing a crucial role in various biochemical processes and material cycles, such as litter decomposition and the formation and breakdown of humus and other organic compounds. They serve as vital indicators of soil fertility (García-Ruiz et al., 2008). The enzymatic activities of cellulase and β-glucosidase are particularly significant in regulating the labile organic carbon fraction within the soil, there by influencing the levels of dissolved organic carbon, readily oxidizable organic carbon, and microbial biomass carbon (Li et al., 2016). The results indicate that the addition of straw markedly enhanced the activities of α-glucosidase, β-glucosidase, β-xylosidase, and catalase. This enhancement is primarily attributed to the provision of abundant energy substrates from exogenous organic matter, which stimulates the metabolic activity of soil microorganisms (Li et al., 2024). Furthermore, enhancements in the soil environment facilitate the transport of photosynthetic products from plants into the soil via root exudates, thereby accelerating enzymatic reactions (Stassen et al., 2021). Meanwhile, the experiment demonstrates that the application of biochar significantly elevated the activities of soil catalase and cellulase. Biochar serves a dual function: it not only protects soil enzyme activity but also promotes microbial proliferation and growth, thereby enhancing metabolic rates an effect advantageous for improving the interaction between enzymes and their substrates (Feng et al., 2023). Additionally, soil cellulase, a complex enzyme responsible for the degradation of cellulose into glucose, exhibited a change in activity that paralleled that of β-glucosidase (BG) following the addition of organic materials. This observation suggests that, within the context of this study, soil β-glucosidase may play a critical role in regulating cellulose decomposition in the soil (Han and He, 2010). It was also observed that the influence of straw on labile carbon was superior to that of biochar, likely due to the rapid decomposition of straw by bacteria and fungi into simpler compounds such as starch, sucrose, and oligosaccharides, resulting in a swift metabolic response (Zhao et al., 2024). Notably, despite significant alterations in the soil carbon-to-nitrogen ratio, the introduction of organic materials did not affect the activity of N-acetyl-β-glucosaminidase (NAG). This insensitivity may be attributed to NAG’s nature as an acidic hydrolytic enzyme, which is less responsive to variations within a narrow neutral pH range (Wang et al., 2025a).

## 5 Conclusions

This 14-year field study demonstrated that the annual application of straw, biochar, and their combination effectively improved soil health and tomato yield under continuous greenhouse cultivation. Structural equation modeling revealed that particulate organic carbon (POC) served as a pivotal regulator, explaining 82% of the variation in tomato yield. Specifically, straw application most prominently enhanced soil labile carbon content and enzyme activities, while biochar amendment significantly elevated soil pH and mineral-associated organic carbon levels. Notably, the combined application of straw and biochar exerted a synergistic effect, which not only promoted soil carbon and nitrogen accumulation but also further improved tomato yield. In summary, this study advances the field by demonstrating that long-term combined application of straw and biochar synergistically improves soil health and tomato yield, with POC identified as a novel mechanistic target for yield enhancement. These insights provide both a theoretical foundation and practical strategy for mitigating continuous cropping obstacles in greenhouse vegetable production.

## Data Availability

The raw data supporting the conclusions of this article will be made available by the authors, without undue reservation.

## Supplementary material

Supplementary Table 1 Basic traits of the tested soils.

Supplementary Table 2 Nutrient content of test materials.

Supplementary Table 3 Amounts of fertilizer used in the experimental treatments.
