# Supplementary material for: Long-term application of straw and biochar improves soil carbon fractions, enzyme activity, and tomato yield under continuous greenhouse cultivation
Source: Front Plant Sci. 2026 Apr 30;17:1808305. doi: 10.3389/fpls.2026.1808305 (PMC13172813; doi:10.3389/fpls.2026.1808305)
Supplement: Supplementary Table 1 — Basic traits of the tested soils. [file Table1.docx]

**Table S1 Basic traits of the tested soils**

| pH | EC  (μs cm^-1^) | TN  (g kg^-1^) | TP  (g kg^-1^) | TK  (g kg^-1^) | AN  (mg kg^-1^) | AP  (mg kg^-1^) | AK  (mg kg^-1^) |
| --- | --- | --- | --- | --- | --- | --- | --- |
| 6.10 | 379 | 1.60 | 3.70 | 29 | 236 | 215 | 654 |

**Table S2 Amount of material added in the test treatment**

|  | Chicken manure  (kg plot^-1^) | Straw  (kg plot^-1^) | Biochar  (kg plot^-1^) |
| --- | --- | --- | --- |
| CK | 4.50 | - | - |
| R | 4.50 | 2.10 | - |
| B | 4.50 | - | 1.10 |
| RB | 4.50 | 2.10 | 1.10 |

**Table S3 Nutrient content of test materials**

|  | pH | EC  (μs cm^-1^) | TN  (g kg^-1^) | TP  (g kg^-1^) | TK  (g kg^-1^) | AN  (mg kg^-1^) | AP  (mg kg^-1^) | AK  (mg kg^-1^) |
| --- | --- | --- | --- | --- | --- | --- | --- | --- |
| Chicken manure | 8.33 | 7430 | 22.20 | 14.90 | 16.60 | 519 | 346 | 138 |
| Straw | - | - | 9.80 | 1.60 | 6.30 | - | - | - |
| Biochar | 9.60 | 190 | 2.53 | 0.78 | 1.68 | 136 | 312 | 3248 |

**Table S4 Amounts of fertilizer used in the experimental treatments**

|  | Urea  (g plot^-1^) | KH_2_PO_4_  (g plot^-1^) | K_2_SO_4_  (g plot^-1^) |
| --- | --- | --- | --- |
| CK | 118.00 | 10.60 | 181.70 |
| R | 80.40 | 6.60 | 141.00 |
| B | 97.50 | 4.00 | 130.80 |
| RB | 60.00 | 0.00 | 90.00 |


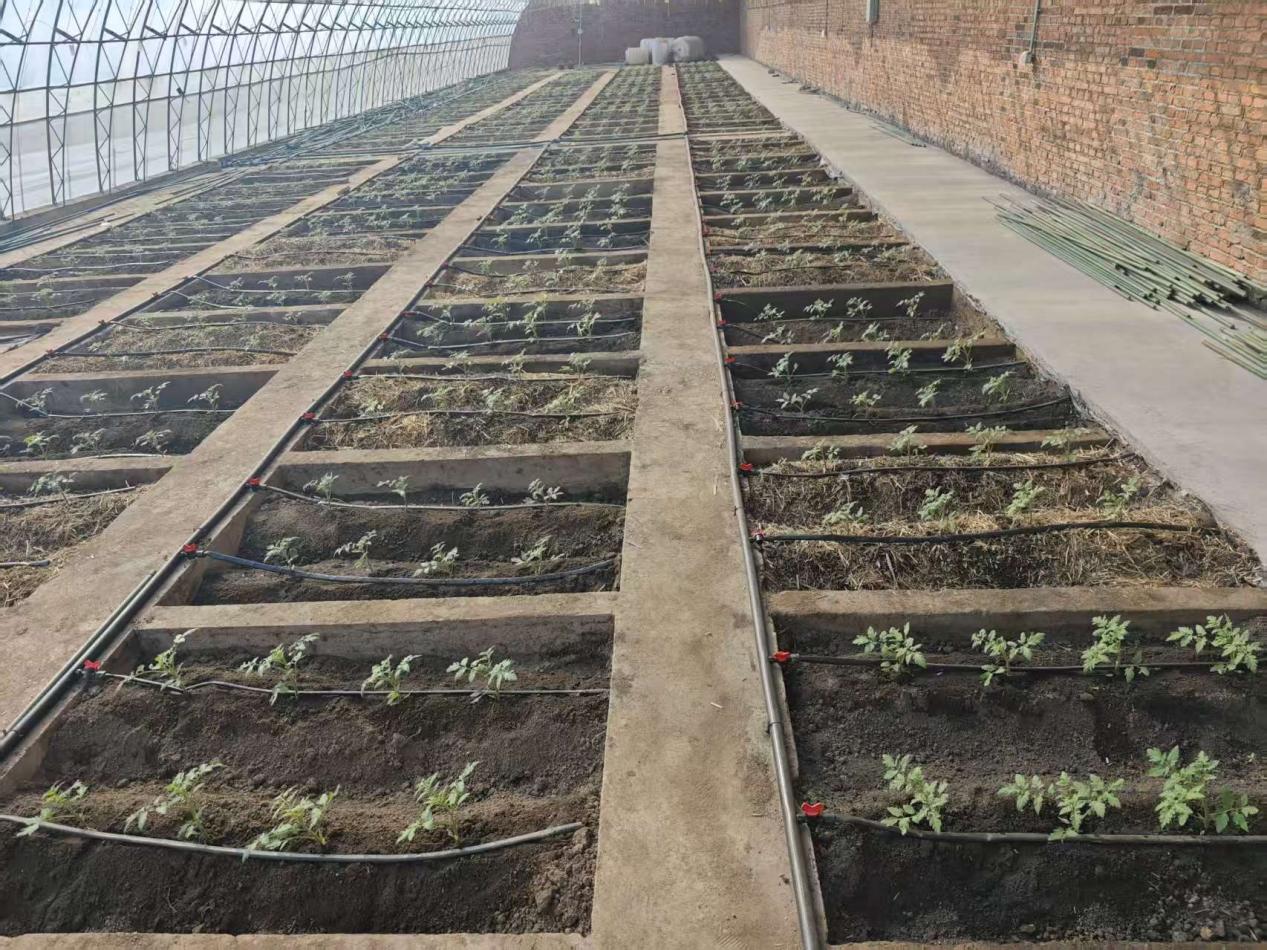


Figure S1 Greenhouse cultivation pool
